# Association of dietary flavonoid intake with incident atherosclerosis: cohort evidence in middle-aged and older adults

**DOI:** 10.3389/fnut.2026.1746512

**Published:** 2026-02-18

**Authors:** Jing Tian, Sicong Wang, Xinyi Chang, Yi Han

**Affiliations:** Department of Critical Care Medicine, The Second Affiliated Hospital of Harbin Medical University, Harbin, China

**Keywords:** all-cause mortality, atherosclerosis, cardiovascular disease, flavonoid, middle-age

## Abstract

**Objective:**

Cardiovascular disease, largely driven by atherosclerosis, remains a leading cause of global morbidity and mortality. This study aimed to investigate the associations of dietary intake of total flavonoids and their subclasses with the risk of incident atherosclerosis, post-atherosclerosis cardiovascular disease and all-cause mortality in a large prospective cohort.

**Methods:**

The study included 207,571 adults without baseline atherosclerosis from the UK Biobank. Risk factors were investigated using a three-level stepwise-adjusted Cox proportional hazards model, alongside subgroup analyses and subclass-specific analyses.

**Results:**

The prevalence of atherosclerosis showed a decreasing trend with increasing quartiles of total flavonoid intake (Q1: 0.75% vs. Q4: 0.48%, *p* < 0.001). In the fully adjusted model, participants in the highest quartile (Q4) of total flavonoid intake exhibited a significantly lower risk of atherosclerosis compared to those in the lowest quartile (Q1) (HR = 0.781, 95% CI: 0.634–0.961, *p* = 0.020). This inverse association persisted across all subgroups, particularly in the age-stratified analysis of individuals aged 55–69 years. Subclass-specific analyses revealed that the protective relationship was primarily driven by flavan-3-ols (HR = 0.660, 95% CI: 0.539–0.807, *p* < 0.001), anthocyanins (HR = 0.816, 95% CI: 0.685–0.971, *p* = 0.022), and flavonols (HR = 0.733, 95% CI: 0.608–0.884, *p* = 0.002). Conversely, total flavonoid intake showed no significant association with cardiovascular disease risk or all-cause mortality following atherosclerosis diagnosis.

**Conclusion:**

Higher consumption of foods rich in flavonoids—such as apples, berries, and tea—was associated with a lower risk of incident atherosclerosis. This inverse association was particularly pronounced among middle-aged and older adults aged 55 to 69. Therefore, incorporating more of these foods into daily diets may be a potential strategy to support cardiovascular health and reduce the burden of subsequent cardiovascular diseases.

## Background

Cardiovascular disease (CVD) ranks among the leading causes of death and disability worldwide (1), with its pathological basis primarily rooted in atherosclerosis. Atherosclerosis is not merely a simple lipid deposition but an active pathological process triggered by endothelial dysfunction and driven by the combined forces of oxidative stress and chronic inflammation (2). Under the influence of risk factors such as hypertension, smoking, and diabetes, vascular endothelial cells endure persistent oxidative stress (3). This leads to reduced nitric oxide (NO) bioavailability and activates pro-inflammatory signaling pathways, thereby promoting monocyte migration, foam cell formation, and the initial establishment of atherosclerotic plaques (4).

In recent years, plant-based dietary patterns have garnered significant attention for their potential role in cardiovascular health (5). Flavonoids, a class of polyphenolic compounds widely present in plant foods such as fruits, vegetables, tea, red wine, and dark chocolate, are considered key bioactive components contributing to these potential benefits (6). Based on chemical structure, flavonoids can be classified into six major subclasses: flavonones, flavones, flavan-3-ols (including proanthocyanidins), flavonols, anthocyanins, and isoflavones (7). Significant differences exist among these subclasses in terms of bioavailability, dietary sources, and biological activity (8).

Multiple epidemiological studies and randomized controlled trials indicate that flavonoid intake is associated with a lower CVD risk. For example, Grassi et al. (2) systematically reviewed the association of tea flavonoids with endothelial function, noting that flavan-3-ols may improve flow-mediated vasodilation by enhancing NO synthesis or inhibiting its degradation, potentially delaying the progression of atherosclerosis. Furthermore, Bondonno et al. (9) observed in the Multi-Ethnic Study of Atherosclerosis that higher flavonoid intake was significantly associated with lower rates of abnormal ankle-brachial index and carotid plaque development, suggesting that flavonoid intake may be inversely associated with atherosclerosis in distinct vascular regions.

Although existing evidence supports the potential of flavonoids in cardiovascular risk reduction, their associations with different stages of atherosclerosis—such as initial onset, progression, and clinical events—remain incompletely understood. Furthermore, the heterogeneous associations among flavonoid subclasses, their differential relations in specific populations, and their link with clinical endpoints require further systematic evaluation.

Therefore, this study, based on the large-scale prospective UK Biobank cohort, aims to systematically investigate the association between total flavonoid and its subclass intake and the occurrence, progression, and all-cause mortality of post-atherosclerosis cardiovascular disease. It will also conduct in-depth analyses of potential differences across various population subgroups to provide scientific evidence to inform dietary guidelines and strategies for cardiovascular health promotion.

## Methods

### Study population

This study is based on the UK Biobank, a large-scale, population-based prospective cohort. This cohort study recruited over 500,000 participants through 22 assessment centers established in England, Scotland, and Wales. At baseline, all participants completed a detailed electronic questionnaire and underwent a series of physical measurements. The study protocol was approved by the National Health Service (NHS) North West Multi-Centre Research Ethics Committee. All participants provided written informed consent prior to enrollment.

**Figure 1:**
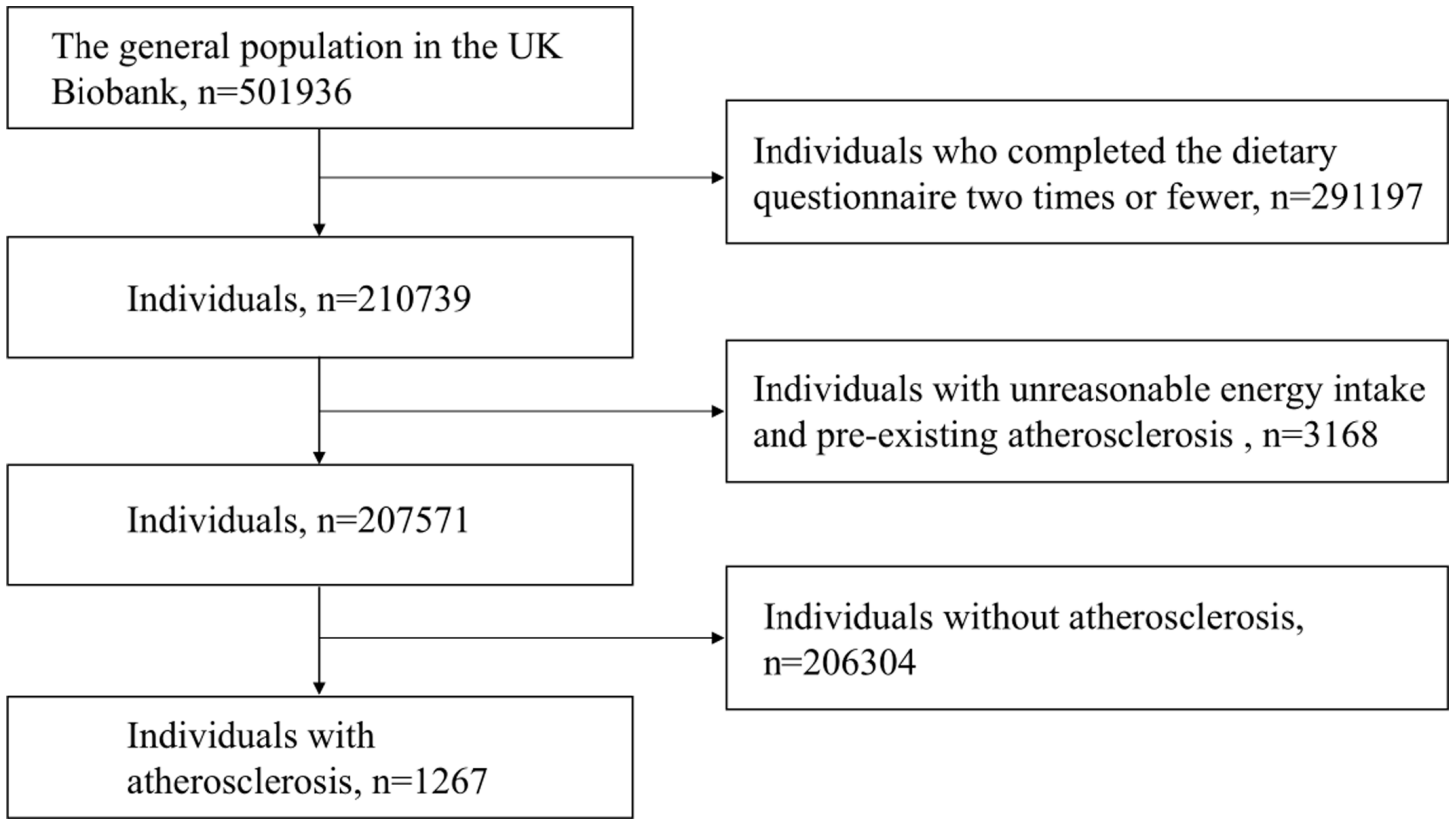
Inclusion and exclusion process for this study.

Participants who completed fewer than two 24-h dietary assessments and had implausible energy intake (males >17,573 kJ or <3,347 kJ and females >14,644 kJ or <2,092 kJ) were excluded from the analysis. Participants with atherosclerosis prior to baseline assessment were also excluded. The relevant exclusion process is shown in Figure 1.

### Assessment of dietary flavonoid intake

Dietary information was collected using the Oxford WebQ, a web-based 24-h dietary recall questionnaire administered at baseline and during four subsequent online follow-ups. Participants reported their consumption of approximately 200 food and beverage items over the previous 24 h. The flavonoid content of food items was estimated using the USDA Database for the Flavonoid Content of Selected Foods (Release 3.1). We matched each food item in the Oxford WebQ to the most appropriate entry in the USDA database. For composite foods, flavonoid content was calculated based on the proportional weight of ingredients. Total flavonoid intake was calculated as the sum of six major subclasses: flavanones, flavones, flavan-3-ols, flavonols, anthocyanins, and isoflavones. To estimate habitual dietary intake and mitigate the effects of day-to-day variation, we calculated the mean flavonoid intake for each participant based on at least two valid 24-h dietary recalls. Based on the distribution of habitual total flavonoid intake within the study population, participants were categorized into quartiles (Q1–Q4) for further analysis, with the lowest quartile (Q1) serving as the reference group.

### Covariates

Socio-demographic, dietary, and lifestyle factors were self-reported during the baseline assessment conducted between 2006 and 2010. Covariates considered in this study included: gender, age at recruitment, education, smoking status, BMI, Townsend Deprivation Index, diabetes, hypertension, whole grain intake, sugar-sweetened beverage intake, processed meat intake, coffee intake, and sodium intake. For more detailed information on how covariates in this study were categorized, see Supplementary Table S1.

### Case ascertainment

The list of codes encompassing atherosclerosis and CVD can be found in Supplementary Table S2. All-cause mortality was determined through linkage with the national death registry, serving as the primary outcome for both the atherosclerosis event analysis and the mortality analysis.

### Outcome ascertainment

Health outcomes were ascertained through linkage to national hospital admission datasets (Hospital Episode Statistics for England, Patient Episode Database for Wales, and Scottish Morbidity Records) and national death registries (NHS Digital).

The primary outcome, incident atherosclerosis, was defined using the International Classification of Diseases, 10th Revision (ICD-10) code I70. To ensure comprehensive case identification, we included diagnoses recorded in both primary and secondary positions.

Secondary outcomes included post-atherosclerosis cardiovascular disease (CVD) and all-cause mortality. Post-atherosclerosis CVD was defined as the first occurrence of major cardiovascular events following the diagnosis of atherosclerosis, specifically including angina pectoris (ICD-10 code I20), acute myocardial infarction (I21), subsequent myocardial infarction (I22), and certain current complications following acute myocardial infarction (I23). A complete list of the specific ICD codes used in this study is provided in Supplementary Table S2.

### Statistical analyses

Participants’ characteristics were summarized for the overall cohort and across quartiles of total flavonoid intake. Continuous variables were presented as mean (standard deviation) or median (interquartile range) based on their distribution, while categorical variables were expressed as frequencies (percentages). Differences across quartiles were assessed using analysis of variance (ANOVA), Kruskal–Wallis tests, or Chi-squared tests, as appropriate.

The associations between flavonoid intake (both total and subclasses) and the risks of atherosclerosis incidence, post-atherosclerosis CVD, and all-cause mortality were evaluated using Cox proportional hazards models. Results were presented as hazard ratios (HRs) and 95% confidence intervals (CIs). Flavonoid intake was analyzed as a categorical variable using quartiles, with the lowest quartile (Q1) serving as the reference group. We constructed three sequential models to increasingly control for potential confounders: Model 1 was adjusted for demographic factors (age and gender). Model 2 further adjusted for socioeconomic, lifestyle, and health factors, including body mass index (BMI), smoking status, education level, Townsend deprivation index, hypertension, and diabetes. Model 3 (the fully adjusted model) additionally controlled for dietary factors, including intakes of sodium, coffee, whole grains, processed meat, and sugar-sweetened beverages. The rationale for this incremental adjustment strategy was to differentiate the influence of non-modifiable biological factors (Model 1) from modifiable lifestyle, socioeconomic, and health conditions (Model 2), and finally to isolate the independent association of flavonoid intake from other dietary habits (Model 3).

Subgroup analyses were performed by stratifying the data according to Gender, Age, BMI (<25 vs. ≥25 kg/m^2^), Smoking status, Education level, and the presence of Hypertension or Diabetes. All statistical analyses were performed using R software (version 4.5.0; R Foundation for Statistical Computing). A two-sided *p*-value of <0.05 was considered statistically significant.

## Results

### Baseline characteristics

A total of 207,571 participants were enrolled in this study and followed for a median of 13.25 years. Table 1 presents the baseline characteristics of the entire study cohort, as well as the characteristics stratified by quartiles of total flavonoid intake. Based on the cut-off values of total flavonoid intake, participants were divided into four groups according to the quartiles of their intake: Q1 (≤58.61 mg/day), Q2 (58.62–109.54 mg/day), Q3 (109.55–203.25 mg/day), and Q4 (≥203.26 mg/day).

**Table 1:** Baseline characteristics of participants according to quartiles of total flavonoid intake.

	Overall, *N* = 207,571	Q1, *N* = 51,893	Q2, *N* = 51,893	Q3, *N* = 51,892	Q4, *N* = 51,893	*p*-value
Age, years	56.1 (7.9)	54.8 (8.2)	56.1 (8.0)	56.4 (7.9)	57.1 (7.5)	<0.001
Gender, *n* (%)						<0.001
Female	114,524 (55.17%)	26,273 (50.63%)	28,279 (54.49%)	29,171 (56.21%)	30,801 (59.35%)	
Male	93,047 (44.83%)	25,620 (49.37%)	23,614 (45.51%)	22,721 (43.79%)	21,092 (40.65%)	
BMI						<0.001
Underweight, (<18.5 kg/m^2^)	1,125 (0.54%)	233 (0.45%)	269 (0.52%)	263 (0.51%)	360 (0.70%)	
Normal, (18.5–25 kg/m^2^)	76,123 (36.78%)	16,044 (31.03%)	18,237 (35.25%)	19,815 (38.28%)	22,027 (42.54%)	
Overweight, (25–30 kg/m^2^)	86,017 (41.56%)	21,871 (42.30%)	21,822 (42.18%)	21,651 (41.83%)	20,673 (39.92%)	
Obese, (≥30 kg/m^2^)	43,715 (21.12%)	13,560 (26.22%)	11,406 (22.05%)	10,028 (19.38%)	8,721 (16.84%)	
Smoking-status, *n* (%)						<0.001
Never	117,124 (56.58%)	28,592 (55.30%)	29,266 (56.57%)	29,364 (56.71%)	29,902 (57.74%)	
Previous	73,660 (35.59%)	17,736 (34.30%)	18,179 (35.14%)	18,784 (36.28%)	18,961 (36.61%)	
Current	16,229 (7.84%)	5,376 (10.40%)	4,293 (8.30%)	3,632 (7.01%)	2,928 (5.65%)	
Education, *n* (%)						<0.001
Unknown	18,827 (9.07%)	5,686 (10.96%)	5,460 (10.52%)	4,105 (7.91%)	3,576 (6.89%)	
College	88,233 (42.51%)	19,282 (37.16%)	20,665 (39.82%)	23,253 (44.81%)	25,033 (48.24%)	
Other levels	100,511 (48.42%)	26,925 (51.89%)	25,768 (49.66%)	24,534 (47.28%)	23,284 (44.87%)	
Townsend deprivation index	−1.6 (2.9)	−1.3 (3.0)	−1.6 (2.9)	−1.7 (2.8)	−1.8 (2.8)	<0.001
Hypertension, *n* (%)	76,383 (36.80%)	19,671 (37.91%)	19,317 (37.22%)	18,714 (36.06%)	18,681 (36.00%)	<0.001
Diabetes, *n* (%)	10,723 (5.17%)	3,370 (6.49%)	2,707 (5.22%)	2,453 (4.73%)	2,193 (4.23%)	<0.001
Sodium intake, mg/day	658.6 (321.9−920.3)	435.2 (228.6–567.8)	482.5 (257.6–640.9)	700.3 (386.7–637.2)	1,016.5 (694.4–1278.8)	<0.001
Coffee intake, cups per day	0.0 (0.0–1.0)	0.0 (0.0–1.0)	0.0 (0.0–1.0)	0.0 (0.0–1.0)	0.0 (0.0–1.0)	<0.001
Whole grains, servings per day	1.5 (0.0, 3.0)	2.0 (1.0, 3.0)	2.1 (1.0, 3.0)	2.0 (1.0, 3.0)	2.0 (0.0, 3.0)	<0.001
Processed meat, servings per day	0.8 (0.2–1.5)	0.6 (0.3–1.6)	0.5 (0.3–1.4)	0.5 (0.3–1.4)	0.6 (0.3–1.4)	<0.001
Sugary drinks Intake, servings per day	0.2 (0.0–0.8)	0.1 (0.0–0.8)	0.0 (0.0–0.8)	0.0 (0.0–0.8)	0.0 (0.0–0.8)	<0.001
Outcome						
Atherosclerosis, *n* (%)	1,267 (0.61%)	388 (0.75%)	323 (0.62%)	305 (0.59%)	251 (0.48%)	<0.001

BMI, body mass index.

Data expressed as median [IQR] or *n* (%), unless otherwise stated.

Compared to those in the lowest quartile of flavonoid intake (Q1, ≤58.61 mg/day), participants in the higher quartiles (Q2–Q4) were more likely to be older, female, and have a normal BMI. The prevalence of obesity, current smoking, and diabetes decreased from Q1 to Q4. Socioeconomically, participants in Q4 had the lowest Townsend Deprivation Index and the highest proportion of college education. Significant differences were also observed in dietary intakes. Sodium intake was highest in Q4. The intake of coffee, whole grains, processed meat, and sugary drinks also varied significantly across quartiles. For the outcome, the prevalence of atherosclerosis showed a decreasing trend across increasing flavonoid intake quartiles (0.75% in Q1, 0.62% in Q2, 0.59% in Q3, and 0.48% in Q4; *p* < 0.001).

### Distribution of flavonoid intake

**Figure 2:**
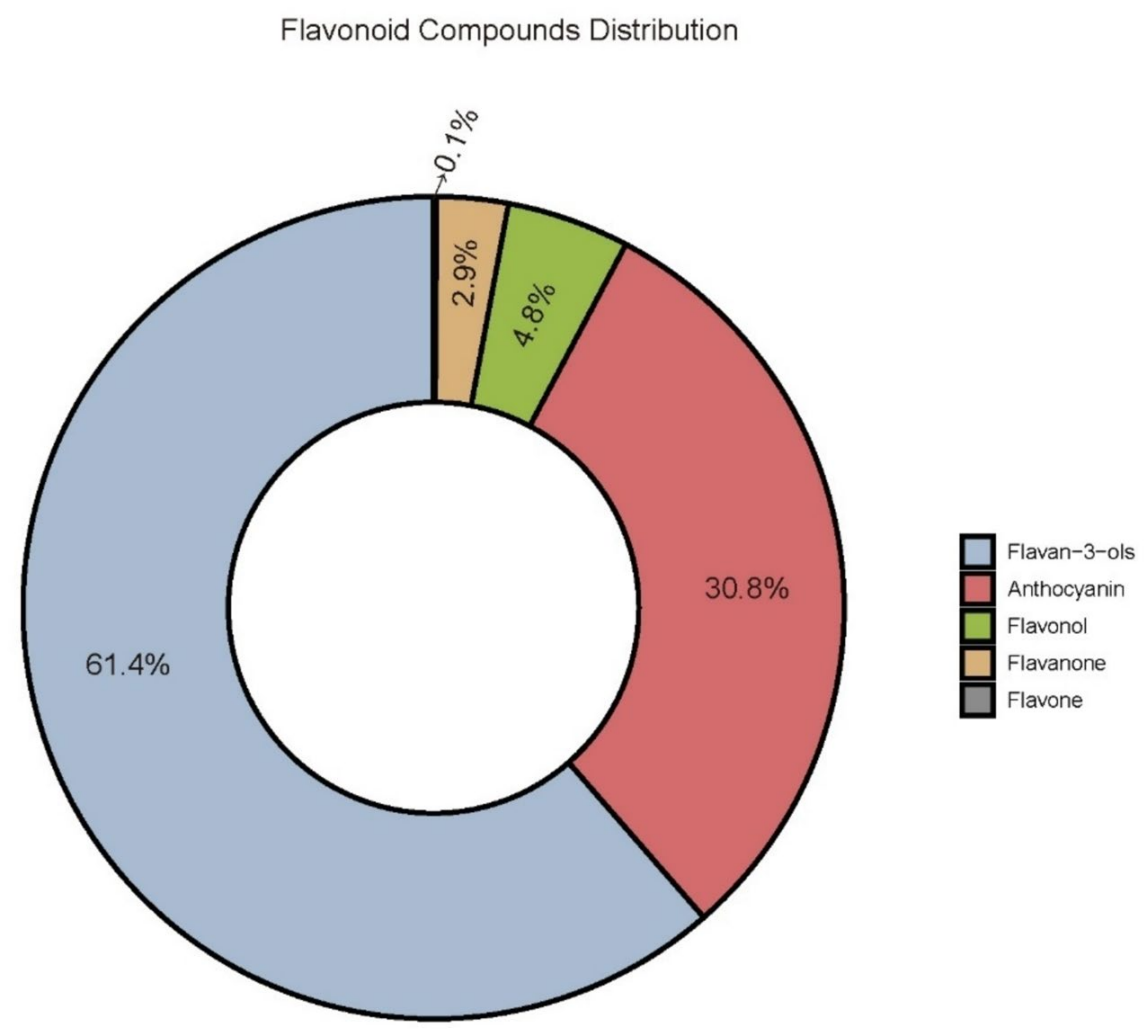
Composition distribution characteristics of dietary flavonoid compounds among participants.

Figure 2 illustrates the compositional distribution of dietary flavonoid intake among the study participants. Flavan-3-ols were the most predominant subclass, accounting for 61.4% of the total intake, with primary dietary sources including tea, apples, and dark chocolate. Anthocyanins constituted the second largest subclass (30.8%), mainly derived from berries, grapes, and red wine. In contrast, flavanones contributed relatively little (2.8%), primarily originating from citrus fruits such as oranges and grapefruits, as well as their juices. Flavones represented the smallest proportion (0.123%), with major food sources including bell peppers, celery, and olives. The remaining portion, approximately 4.877%, consisted of other unclassified flavonoid compounds, reflecting the diversity of dietary flavonoid sources.

**Figure 3:**
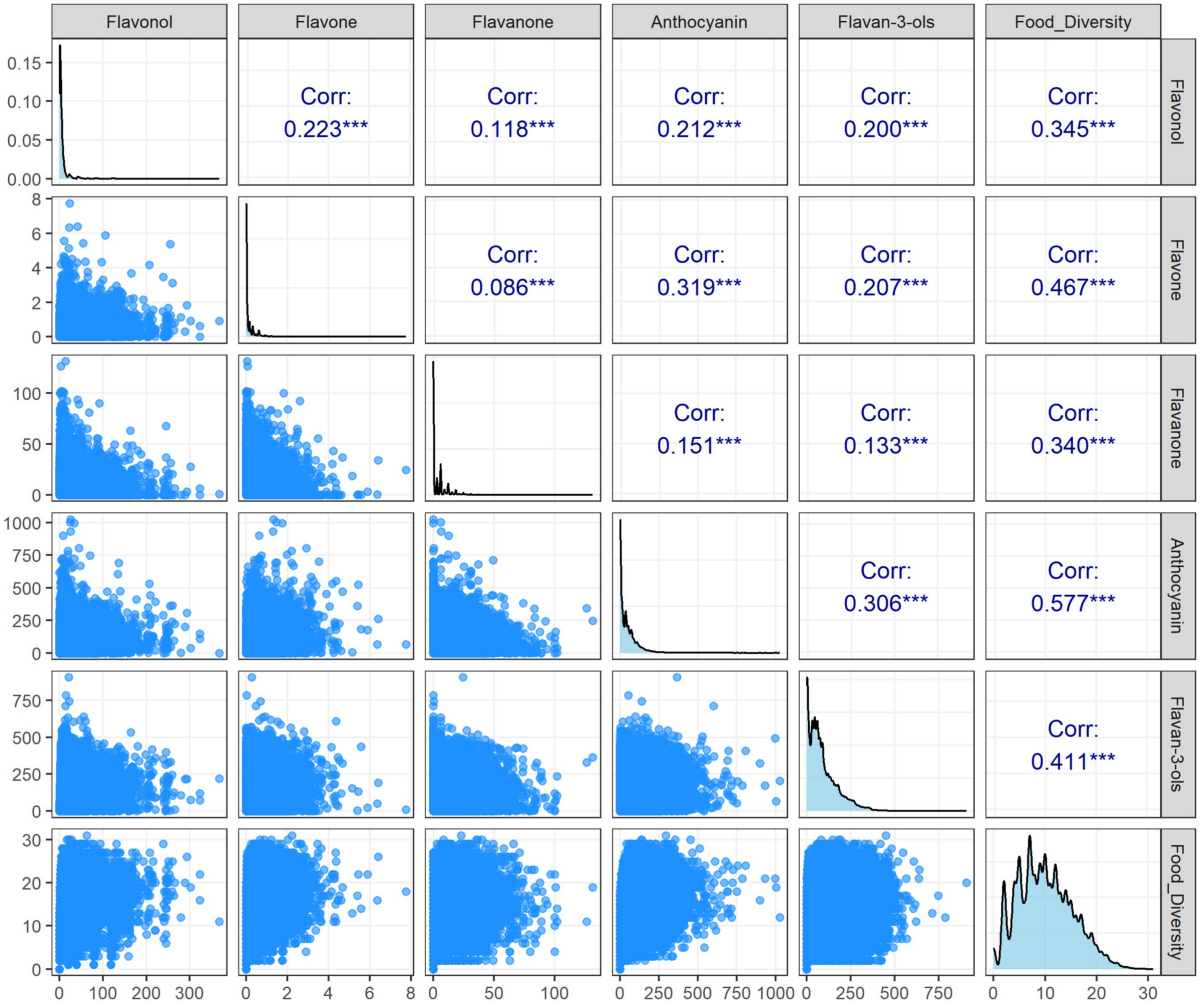
Intake distribution of individual dietary flavonoid subclasses and their correlations with food diversity among participants.

As shown in Figure 3, Flavan-3-ols predominated in dietary flavonoid intake and exhibited significant individual variability. They were followed in sequence by Anthocyanins, Flavonols, Flavanones, and Flavones, presenting a decreasing trend in intake and a progressively concentrated data distribution. Further correlation analysis revealed that the strength of the association between flavonoid intake and food diversity varied by subclass. The strongest association was observed for Flavan-3-ols (*R* = 0.41), while Anthocyanins (*R* = 0.31) and Flavonols (*R* = 0.22) showed moderate associations. In contrast, the weakest associations were seen for Flavones (*R* = 0.09) and Flavanones (*R* = 0.15).

### Characteristics of individuals with atherosclerosis

A total of 1,267 patients with atherosclerosis were included in this subgroup, with a median follow-up time of 8.8 years. The mean age of patients was 61.8 years, with a higher proportion of males (68.19%). Compared with the lowest intake group (Q1), patients in the higher intake groups (Q3 and Q4) were older. Furthermore, the proportion of current smokers decreased significantly from 29.11% in the Q1 group to 17.35% in the Q4 group. The prevalence of diabetes was also highest in the Q1 group (24.29%) and decreased to the lowest level in the Q4 group (16.09%). Regarding educational level, the Q4 group had the highest proportion of patients with a college degree. In terms of dietary factors, sodium intake was highest in the Q4 group. Significant differences were also observed in the intakes of coffee, whole grains, processed meat, and sugary drinks across the groups. Although no statistically significant difference in all-cause mortality was observed among atherosclerotic patients across the groups (*p* = 0.342), the Q4 group had the lowest all-cause mortality (30.68%) (see Table 2).

**Table 2:** Baseline characteristics and all-cause mortality of atherosclerotic patients’ subgroup.

	Overall, *N* = 1,267	Q1, *N* = 317	Q2, *N* = 317	Q3, *N* = 316	Q4, *N* = 317	*p*-value
Age, years	61.8 (5.9)	60.7 (6.2)	61.8 (6.0)	62.4 (5.5)	62.4 (5.7)	<0.001
Gender, *n* (%)						0.383
Female	403.0 (31.81%)	90.0 (28.39%)	99.0 (31.23%)	109.0 (34.49%)	105.0 (33.12%)	
Male	864.0 (68.19%)	227.0 (71.61%)	218.0 (68.77%)	207.0 (65.51%)	212.0 (66.88%)	
BMI						0.288
Underweight, (<18.5 kg/m^2^)	10.0 (0.79%)	1.0 (0.32%)	4.0 (1.27%)	3.0 (0.96%)	2.0 (0.63%)	
Normal, (18.5–25 kg/m^2^)	302.0 (24.01%)	74.0 (23.57%)	63.0 (20.06%)	77.0 (24.52%)	88.0 (27.85%)	
Overweight, (25–30 kg/m^2^)	552.0 (43.88%)	132.0 (42.04%)	156.0 (49.68%)	133.0 (42.36%)	131.0 (41.46%)	
Obese, (≥30 kg/m^2^)	394.0 (31.32%)	107.0 (34.08%)	91.0 (28.98%)	101.0 (32.17%)	95.0 (30.06%)	
Smoking-status, *n* (%)						0.002
Never	385.0 (30.46%)	80.0 (25.32%)	101.0 (31.96%)	100.0 (31.75%)	104.0 (32.81%)	
Previous	611.0 (48.34%)	144.0 (45.57%)	146.0 (46.20%)	163.0 (51.75%)	158.0 (49.84%)	
Current	268.0 (21.20%)	92.0 (29.11%)	69.0 (21.84%)	52.0 (16.51%)	55.0 (17.35%)	
Education, *n* (%)						0.017
Unknown	267.0 (21.07%)	86.0 (27.13%)	71.0 (22.40%)	62.0 (19.62%)	48.0 (15.14%)	
College	340.0 (26.84%)	73.0 (23.03%)	87.0 (27.44%)	85.0 (26.90%)	95.0 (29.97%)	
Other levels	660.0 (52.09%)	158.0 (49.84%)	159.0 (50.16%)	169.0 (53.48%)	174.0 (54.89%)	
Townsend deprivation index	−1.0 (3.1)	−0.6 (3.1)	−1.1 (3.2)	−1.2 (2.9)	−1.0 (3.1)	0.134
Hypertension, *n* (%)	1,026.0 (80.98%)	261.0 (82.33%)	253.0 (79.81%)	254.0 (80.38%)	258.0 (81.39)	0.857
Diabetes, *n* (%)	236.0 (18.63%)	77.0 (24.29%)	48.0 (15.14%)	60.0 (18.99%)	51.0 (16.09%)	0.032
Sodium intake, mg per day	629.1 (295.9–858.8)	422.3 (224.4–559.1)	446.2 (263.3–641.4)	630.8 (368.1–948.2)	1,017.1 (696.2–1391.5)	<0.001
Coffee intake, cups per day	0.3 (0.0–1.0)	0.0 (0.1–0.8)	0.1 (0.1–0.8)	0.1 (0.0–1.0)	0.1 (0.1–1.0)	<0.001
Whole grains, servings per day	1.4 (0.0, 3.0)	1.3 (1.0, 2.0)	1.4 (1.0, 2.0)	1.5 (1.0, 3.0)	1.5 (1.0, 3.0)	0.001
Processed meat, servings per day	0.6 (0.3–1.6)	0.2 (0.1–1.6)	0.2 (0.1–1.6)	0.3 (0.1–1.5)	0.3 (0.1–1.6)	<0.001
Sugary drinks intake, servings per day	0.5 (0.3–1.4)	0.5 (0.3–1,5)	0.5 (0.3–1.4)	0.4 (0.2–1.4)	0.4 (0.2–1.4)	0.003
Outcome						
All-cause mortality, *n* (%)	426 (33.62%)	126 (32.47%)	121 (37.46%)	102 (33.44%)	77 (30.68%)	0.342

BMI, body mass index.

Data expressed as median [IQR] or *n* (%), unless otherwise stated.

### Risk of disease occurrence

Table 3 presents the associations between total dietary flavonoid intake and three key health outcomes using Cox proportional hazards models, including the risk of atherosclerosis incidence, post-atherosclerosis CVD risk, and all-cause mortality in the atherosclerotic population. Regarding atherosclerosis risk, the minimally adjusted Model 1 showed a progressive decrease in HRs with increasing flavonoid intake, with Q2 at HR = 0.782 (95% CI 0.667–0.917), Q3 at HR = 0.711 (95% CI 0.604–0.836), and Q4 at HR = 0.582 (95% CI 0.489–0.691). This inverse association persisted for the Q4 group even after further adjustment for confounding factors such as smoking and hypertension in Model 2, and additional incorporation of dietary factors in Model 3. In contrast, for CVD risk among participants with established atherosclerosis, no statistically significant association was observed at any intake level after full adjustment for dietary factors in Model 3. Furthermore, across all three progressively adjusted models, total dietary flavonoid intake showed no statistically significant association with all-cause mortality.

**Table 3:** Risk of atherosclerosis incidence, cardiovascular disease, and all-cause mortality by quartile of total flavonoid intake.

	Model 1		Model 2		Model 3	
Quantity of total dietary flavonoid intake	HR (95% CI)	*p*-value	HR (95% CI)	*p*-value	HR (95% CI)	*p*-value
Atherosclerosis incidence						
Q1	—	—	—	—	—	—
Q2	0.782 (0.667–0.917)	0.003	0.850 (0.724–0.997)	0.046	0.873 (0.743–1.025)	0.097
Q3	0.711 (0.604–0.836)	<0.001	0.819 (0.695–0.965)	0.017	0.864 (0.728–1.026)	0.096
Q4	0.582 (0.489–0.691)	<0.001	0.692 (0.581–0.823)	<0.001	0.781 (0.634–0.961)	0.020
Cardiovascular disease incidence						
Q1	—	—	—	—	—	—
Q2	0.730 (0.598–0.890)	0.002	0.780 (0.638–0.954)	0.016	0.790 (0.645–0.967)	0.022
Q3	0.783 (0.642–0.954)	0.015	0.816 (0.668–0.997)	0.047	0.834 (0.678–1.026)	0.086
Q4	0.825 (0.679–1.003)	0.054	0.872 (0.715–1.064)	0.178	0.929 (0.728–1.186)	0.555
All-cause mortality						
Q1	—	—	—	—	—	—
Q2	0.952 (0.729–1.244)	0.720	1.057 (0.807–1.385)	0.686	1.037 (0.791–1.363)	0.794
Q3	0.870 (0.663–1.141)	0.314	0.941 (0.716–1.239)	0.667	0.904 (0.681–1.223)	0.486
Q4	0.830 (0.632–1.091)	0.183	0.925 (0.701–1.221)	0.582	0.879 (0.629–1.227)	0.448

Model 1: Age, Gender, BMI.

Model 2: Age, Gender, BMI, Townsend Deprivation Index, Smoking-status, Education, Hypertension, Diabetes.

Model 3: Age, Gender, BMI, Townsend Deprivation Index, Smoking-status, Education, Hypertension, Diabetes, Sodium intake, Coffee intake, Whole grains, Processed meat, Sugary drinks intake.

To further clarify the specific sources of flavonoid protective effects, we conducted a refined Cox proportional hazards analysis at the flavonoid subclass level in the Supplementary materials. This explored the associations between flavan-3-ols, anthocyanins, flavanols, flavones, and flavones with the risk of post-atherosclerosis cardiovascular disease onset, and all-cause mortality. Adjustment strategies were consistent with Table 3 in the main text (Model 1: age, gender, BMI; Model 2: added socioeconomic status, lifestyle factors, and underlying diseases; Model 3: Further adjusted for dietary factors). Detailed data are presented in Supplementary Table S3. Discovery of flavan-3-ols and flavonols represent the core subclass of flavonoids exerting protective effects against atherosclerosis. Anthocyanins and flavanols also demonstrate independent protective actions, whereas the effects of flavones and flavonones lack robustness. Only anthocyanins show a weak protective effect on CVD risk following atherosclerosis. None of the subclasses exhibit significant associations with all-cause mortality.

### Different sub-group analysis

**Figure 4:**
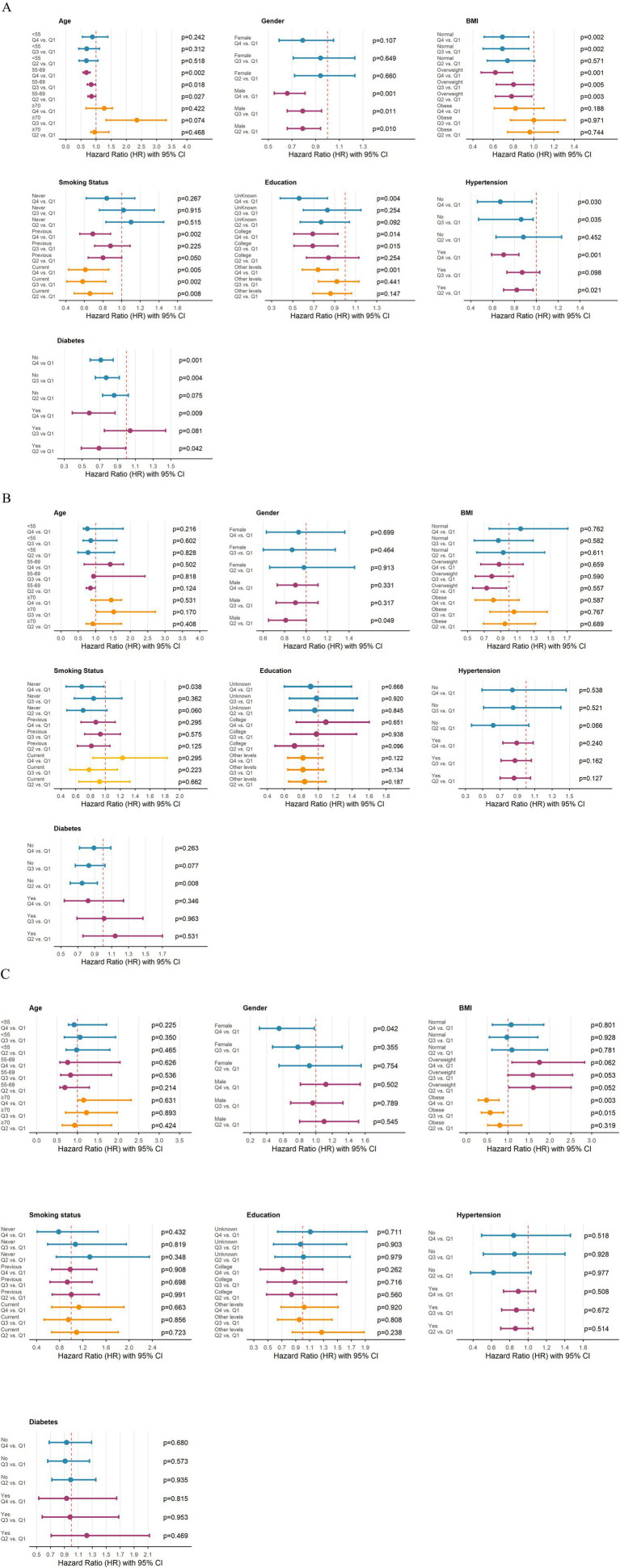
Subgroup analyses of the association between total dietary flavonoid intake and the risk of atherosclerosis incidence, cardiovascular disease, and all-cause mortality. **(A)** The risk of atherosclerosis incidence; **(B)** the risk of cardiovascular disease incidence; **(C)** the risk of all-cause mortality.

As shown in Figure 4A, the protective association between flavonoid intake and the risk of developing atherosclerotic disease was consistent across all subgroups, with this trend being particularly evident in the age-stratified analysis. Specifically, in the 55–69 age group, flavonoid intake was associated with a reduced risk of atherosclerotic disease. Furthermore, this association remained robust across other stratification factors, including male sex, overweight status, current smoking, and coexisting hypertension or diabetes, indicating that higher flavonoid intake exerts a protective effect against the risk of developing atherosclerotic disease. In contrast, Figure 4B reveals heterogeneity in the association between flavonoid intake and cardiovascular disease risk following atherosclerosis across different subgroups. The significant protective effect of flavonoid intake was observed only in specific populations, including males and individuals who had never smoked; no significant protective association was noted in other subgroups. Furthermore, the analysis of all-cause mortality risk in Figure 4C indicates that flavonoid intake was not significantly associated with all-cause mortality in any subgroup, regardless of stratification by sex, age, BMI, smoking status, education level, or presence of hypertension or diabetes.

## Discussion

This study utilized a cohort of 207,571 adults without baseline atherosclerosis from the UK Biobank, employing a three-level stepwise confounder-adjusted Cox proportional hazards model alongside subgroup analyses to further explore the independent associations of flavonoid subclasses. It systematically assessed the association between total dietary flavonoids and their subclasses with three key outcomes: the risk of atherosclerosis onset, the risk of post-atherosclerosis cardiovascular disease onset, and all-cause mortality. Research findings indicate that total dietary flavonoid intake exhibits a significant inverse association with the risk of developing atherosclerosis. According to the fully adjusted model (Model 3), participants in the highest quartile (Q4, ≥203.26 mg/day) of total dietary flavonoid intake exhibited a significantly lower risk of atherosclerosis (HR = 0.781, 95% confidence interval: 0.634–0.961, *p* = 0.020) was significantly lower than that in the lowest quartile (Q1, ≤58.61 mg/day). Subgroup analyses further confirmed that this inverse association remained consistent across different genders, BMI strata, and among individuals with hypertension or diabetes—particularly pronounced in adults aged 55–69 years. In contrast, after adjusting for dietary factors, the association between total flavonoid intake and the risk of cardiovascular disease following atherosclerosis was no longer statistically significant. Furthermore, no significant association was observed between total dietary flavonoid intake and all-cause mortality. The inverse association of flavonoids with atherosclerosis exhibited significant subclass specificity. Flavan-3-ols emerged as the core effect subgroup (Q4 vs. Q1: HR = 0.660, 95% CI: 0.539–0.807, *p* < 0.001), whereas anthocyanins (Q4 vs. Q1: HR = 0.816, 95% CI: 0.685–0.971, *p* = 0.022) and flavonols (Q4 vs. Q1: HR = 0.733, 95% CI: 0.608–0.884, *p* = 0.002) also exhibited independent inverse associations. Conversely, the effects of flavones and flavanones were no longer significant after multivariate adjustment, indicating fundamental differences in the associations with health outcomes of distinct flavonoid subclasses.

CVD is the leading cause of death and disability worldwide, with its pathological basis primarily rooted in atherosclerosis (10). Atherosclerosis is not merely a simple lipid deposition but an active pathological process initiated by endothelial dysfunction and driven by the combined forces of oxidative stress and chronic inflammation (11). Under the influence of risk factors such as hypertension, smoking, and diabetes, vascular endothelial cells endure persistent oxidative stress. This reduces the bioavailability of NO and activates pro-inflammatory signaling pathways (e.g., NF-κβ) (12). Endothelial cells within the body enter a pro-inflammatory state, increasing vascular permeability. Lipids infiltrate and oxidize, while monocytes chemotactically migrate, adhere, and differentiate into macrophages. These phagocytose oxidized lipids to form foam cells, ultimately establishing the rudiments of atherosclerotic plaques (13, 14). Unstable atherosclerotic plaques may rupture, leading to thrombus formation and blood flow interruption, which can result in serious cardiovascular events such as myocardial infarction and stroke. Previous reports indicate that atherosclerosis is a primary cause of mortality and morbidity (15). Therefore, inhibiting endothelial dysfunction, oxidative stress, and chronic inflammation at their source is a key strategy for potentially delaying the onset and progression of atherosclerosis (16), thereby potentially reducing the overall burden of CVD.

Against this backdrop, this study may hold some significance for public health. The risk of developing atherosclerosis gradually decreases with increasing total dietary flavonoid intake. After adjusting for covariates, the highest intake group showed a significant 21.9% lower risk compared to the lowest intake group (HR = 0.781, 95% CI: 0.634–0.961, *p* = 0.020), confirming the independent association between flavonoid intake and atherosclerosis development. Furthermore, flavan-3-ols, the predominant subclass accounting for 61.4% of total flavonoid intake, emerged as the primary contributor to this association. The high-intake group exhibited a 34.0% lower risk compared to the lowest group (HR = 0.660, 95% CI: 0.539–0.807, *p* < 0.001); Anthocyanins (HR = 0.816, 95% CI: 0.685–0.971, *p* = 0.022) and flavonols (HR = 0.733, 95% CI: 0.608–0.884, *p* = 0.002) also demonstrated independent inverse associations. Furthermore, subgroup analyses confirmed that the inverse association of total dietary flavonoids with atherosclerosis remained consistent across different genders, ages, BMIs, smoking statuses, and hypertension/diabetes statuses. This suggests that increasing flavonoid intake may not be limited to specific low-risk populations but represents a broadly applicable dietary strategy for risk reduction. Its benefits remain consistent across diverse demographic groups, particularly offering a practical dietary intervention direction for individuals with coexisting risk factors such as hypertension and diabetes.

The observed associations of flavonoids are deeply rooted in their bioactivity against the core mechanisms of atherosclerosis. The negative correlation observed in this study can be rationally explained at the molecular and cellular levels. First, improving endothelial function is a key component of flavonoid action. Randomized controlled trials have confirmed that flavan-3-ols, in particular, significantly promote NO release and restore vasodilatory function (12). In a study by Dalgaard et al. (17), flavonoid intake was found to be negatively correlated with atherosclerotic CVD, with the strongest association observed in peripheral atherosclerotic disease. Our study corroborates this finding, identifying Flavan-3-ols as the core subclass driving the inverse associations (Q4 vs. Q1: HR = 0.660). This aligns with Flavan-3-ols being the most abundant dietary flavonoid in humans and the subclass with the strongest evidence for endothelial protection. Second, flavonoids are potent antioxidants and anti-inflammatory agents. They neutralize reactive oxygen species (ROS) and mitigate vascular wall inflammation by upregulating the Nrf2 pathway and suppressing the NF-κβ pathway (18, 19). We found that both anthocyanins and flavanols exhibit independent inverse associations. Previous studies indicate that anthocyanin metabolites reduce IL-6 and vascular cell adhesion molecule-1 (VCAM-1) in human vascular endothelial cells stimulated by oxidized low-density lipoprotein cholesterol or cluster of differentiation 40 (CD40) ligand (20). Furthermore, higher anthocyanin and flavanol intake correlates with reduced systemic inflammation levels. For example, a study by Cassidy et al. (21) showed that intakes of flavonols and anthocyanins were significantly inversely associated with total inflammatory score (IS): flavonols intake in the highest quintile was 42% lower than that in the lowest quintile (−0.72, P for trend = 0.01), while anthocyanin intake in the highest quintile showed a 73% reduction compared to the lowest quintile (−1.48, P for trend < 0.001), with anthocyanins exhibiting superior anti-inflammatory potential.

The associations effects of flavonoids appear to be disease stage-specific. We found that the association between flavonoid intake and CVD risk after atherosclerosis weakens after adjusting for dietary factors, while showing no significant association with all-cause mortality. This result suggests that the primary “target” of flavonoids may lie in the initiation and early progression stages of atherosclerosis. During this phase, flavonoids may help delay or mitigate the formation of initial lesions by improving endothelial function and suppressing oxidative stress and inflammation (22, 23). However, once the disease progresses to advanced stages, where complex necrotic cores and calcification form within plaques, stability becomes more dependent on mechanical structure and acute inflammatory responses (24, 25). At this point, dietary flavonoid intervention alone may struggle to reverse established severe lesions or prevent acute complications. Similarly, Parmenter et al. (26) found that a high-flavonoid diet showed no association with mortality risk in CVD populations but was associated with a lower CVD incidence risk, consistent with our findings. Furthermore, this study revealed the “heterogeneity” within the flavonoid family through subclass analysis. While total flavonoids demonstrated inverse associations, these were primarily driven by core subclasses including flavan-3-ols, anthocyanins, and flavanones. The effects of flavone and flavonol, however, ceased to be significant after adjusting for multiple factors. This may be attributed to their low dietary prevalence, differences in bioavailability, or inherently weaker biological activity. This finding may suggest that in future dietary guidance or nutritional intervention studies, “flavonoids” should not be treated as a homogeneous group. Instead, greater emphasis should be placed on those subclasses with clearly demonstrated strong associations and their primary dietary sources.

## Limitations

This study has several limitations. First, dietary intake was assessed using the Oxford WebQ online 24-h dietary recall questionnaire, which may not accurately reflect long-term dietary habits, particularly for infrequently consumed flavonoid-rich foods. Additionally, the temporal discrepancy between baseline covariate collection and dietary assessment may introduce exposure misclassification, although we enhanced accuracy by restricting analyses to participants completing ≥2 assessments with adequate energy intake. Second, flavonoid intake estimates carry uncertainty: Food grouping (e.g., combining different “berries”) necessitates the use of averages, and the questionnaire struggles to fully capture the impact of cooking processing on flavonoid content. Furthermore, the Oxford WebQ food database does not perfectly match the USDA database, and these factors may collectively underestimate the true association between flavonoids and health outcomes to some extent. Although Oxford WebQ demonstrated reasonable validity against objective biomarkers, recall bias, over- or under-reporting cannot be entirely ruled out (27). Third, regarding outcome ascertainment, cases of atherosclerosis were identified primarily through linkage to hospital inpatient records and death registries using ICD-10 codes (I70). While this ensures high specificity for clinically significant disease, it may miss early-stage, subclinical, or less severe cases managed exclusively in primary care settings. Consequently, the incidence rates reported in our study may underestimate the true burden of atherosclerosis in the population. Finally, as an observational study, although we adjusted for a wide range of potential confounders including socioeconomic status and lifestyle factors (e.g., smoking, BMI), residual confounding cannot be fully excluded. It is possible that high flavonoid intake serves as a marker for a generally healthier lifestyle or unmeasured behaviors that contribute to the observed cardiovascular benefits.

## Conclusion

This study suggests that higher dietary flavonoid intake, particularly from foods rich in flavan-3-ols, anthocyanins, and flavonols, is associated with a significantly lower risk of incident atherosclerosis. This inverse association was particularly pronounced in middle-aged and older adults aged 55 to 69. Moreover, incorporating common flavonoid-rich foods—such as apples, berries, and tea—into daily diets may represent a valuable strategy to support cardiovascular health and potentially mitigate the risk of atherosclerosis.

## Data Availability

The original contributions presented in the study are included in the article/Supplementary material, further inquiries can be directed to the corresponding author.
