# Supplementary material for: Association of dietary flavonoid intake with incident atherosclerosis: cohort evidence in middle-aged and older adults
Source: Front Nutr. 2026 Feb 18;13:1746512. doi: 10.3389/fnut.2026.1746512 (PMC12956777; doi:10.3389/fnut.2026.1746512)
Supplement: Supplementary file 1 [file Table_1.docx]

Table S1. Covariate coding.

| Variables | Categorisation | UK Biobank variable description and data-field ID |
| --- | --- | --- |
| Demographics |  |  |
| Age | Age at entry (continuous variable) | Age at recruitment (ID: 21022) |
| Gender | Female; Male | Gender (ID: 31) |
| BMI | Underweight (<18.5 kg/m^2^); Healthy weight (18.5-25 kg/m^2^); Overweight (25-30 kg/m^2^); Obese (≥30 kg/m^2^); Unknown/Missing | BMI (ID: 21001) |
| Smoking status | Never; Previous; Current; Unknown/Missing | Smoking status (ID: 20116) |
| Education | Low: CSEs or equivalent, O levels/GCSEs or equivalent; Medium: A levels/AS levels or equivalent, NVQ or HND or HNC or equivalent; High: College or University degree, other professional qualifications eg: nursing, teaching; Unknown/Missing | Qualifications (ID: 6138) |
| Townsend deprivation index | Quintiles from least to most deprived; Unknown/ Missing | Townsend deprivation index (ID: 22189) |
| Hypertension | No; Yes | Hypertension: ICD 10 (I10, I15) |
| Diabetes | No; Yes | Diabetes: ICD10 (E11) |
| Coffee intake | Number of servings reported yesterday | Diet by 24-hour recall (category ID: 100090) |
| Wholegrain intake | Number of servings reported yesterday | Diet by 24-hour recall (category ID: 100090) |
| Sugar-sweetened beverage intake | Number of servings reported yesterday | Diet by 24-hour recall (category ID: 100090) |
| Sodium Intake | Continuous | Diet by 24-hour recall (category ID: 100090) |
| Processed meat | Continuous | Diet by 24-hour recall (category ID: 100090) |

Table S2. Disease Codes and Names.

| Name of Disease | Source | Code |
| --- | --- | --- |
| Atherosclerosis | ICD 10 | I70 |
| Cardiovascular disease |  |  |
| Angina pectoris | ICD 10 | I20 |
| Acute myocardial infarction | ICD 10 | I21 |
| Subsequent myocardial infarction | ICD 10 | I22 |
| Certain current complications following acute myocardial infarction | ICD 10 | I23 |

Table S3. Correlation analysis between individual dietary flavonoid subclass intake and the risk of atherosclerosis onset, cardiovascular disease following atherosclerosis, and all-cause mortality.

|  | Model 1 | | Model 2 | | Model 3 | |
| --- | --- | --- | --- | --- | --- | --- |
| Atherosclerosis incidence |  |  |  |  |  |  |
| Quantity of total dietary flavonoid intake | HR (95%CI) | p-value | HR (95%CI) | p-value | HR (95%CI) | p-value |
| Flavan-3-ols |  |  |  |  |  |  |
| Q1 | - | - | - | - | - | - |
| Q2 | 0.645 (0.525-0.793) | <0.001 | 0.706 (0.574 -0.869) | <0.001 | 0.721 (0.586-0.888) | <0.001 |
| Q3 | 0.648 (0.538-0.781) | <0.001 | 0.751 (0.623-0.906) | 0.027 | 0.780 (0.645-0.942) | 0.001 |
| Q4 | 0.489 (0.408-0.586) | <0.001 | 0.601 (0.501-0.722) | <0.001 | 0.660 (0.539-0.807) | <0.001 |
| Flavonols |  |  |  |  |  |  |
| Q1 | - | - | - | - | - | - |
| Q2 | 0.667 (0.567-0.786) | <0.001 | 0.753 (0.639 -0.887) | 0.001 | 0.766 (0.649-0.904) | 0.001 |
| Q3 | 0.649 (0.55 -0.763) | <0.001 | 0.742 (0.629 -0.875) | <0.001 | 0.769 (0.649-0.913) | 0.003 |
| Q4 | 0.608 (0.516-0.717) | <0.001 | 0.685 (0.579-0.809) | <0.001 | 0.733 (0.608-0.884) | 0.002 |
| Flavanones |  |  |  |  |  |  |
| Q1 | - | - | - | - | - | - |
| Q2 | 0.742 (0.572-0.961) | 0.024 | 0.838 (0.646-1.087) | 0.184 | 0.836 (0.645-1.085) | 0.178 |
| Q3 | 0.821 (0.683-0.988) | 0.037 | 0.931 (0.773-1.122) | 0.454 | 0.933 (0.775-1.124) | 0.464 |
| Q4 | 0.804 (0.683-0.948) | 0.009 | 0.962 (0.815-1.136) | 0.649 | 1.028 (0.865-1.221) | 0.758 |
| Flavones |  |  |  |  |  |  |
| Q1 | - | - | - | - | - | - |
| Q2 | 0.926 (0.480-1.785) | 0.817 | 0.969 (0.503-1.869) | 0.926 | 1.003 (0.518-1.940) | 0.184 |
| Q3 | 0.911 (0.532-1.530) | 0.631 | 0.932 (0.843-1.342) | 0.463 | 0.985 (0.793-1.764) | 0.282 |
| Q4 | 0.803 (0.569-1.233) | 0.922 | 0.920 (0.859-1.454) | 0.830 | 0.940 (0.764-1.330) | 0.397 |
| Anthocyanins |  |  |  |  |  |  |
| Q1 | - | - | - | - | - | - |
| Q2 | 0.627 (0.530-0.743) | <0.001 | 0.736 (0.621-0.873) | <0.001 | 0.749 (0.631-0.889) | 0.001 |
| Q3 | 0.658 (0.559-0.774) | <0.001 | 0.771 (0.654- 0.909) | 0.002 | 0.792 (0.670-0.936) | 0.006 |
| Q4 | 0.640 (0.544-0.753) | <0.001 | 0.778 (0.660-0.917) | 0.003 | 0.816 (0.685-0.971) | 0.022 |
| Cardiovascular disease incidence | HR (95%CI) | p-value | HR (95%CI) | p-value | HR (95%CI) | p-value |
| Flavan-3-ols |  |  |  |  |  |  |
| Q1 | - | - | - | - | -- |  |
| Q2 | 0.954 (0.786-1.156) | 0.628 | 0.969 (0.790-0.176) | 0.750 | 1.010 (0.824-1.239) | 0.922 |
| Q3 | 0.781 (0.636-0.959) | 0.018 | 0.819(0.664-1.009) | 0.061 | 0.881 (0.705-1.100) | 0.264 |
| Q4 | 0.956 (0.784-1.164) | 0.651 | 0.999 (0.818-1.220) | 0.992 | 1.163 (0.899-1.504) | 0.249 |
| Flavonols |  |  |  |  |  |  |
| Q1 | - | - | - | - | - | - |
| Q2 | 0.998 (0.817-1.220) | 0.985 | 0.987 (0.806-1.208) | 0.895 | 1.022 (0.833-1.255) | 0.832 |
| Q3 | 0.972(0.796-1.186) | 0.778 | 1.008 (0.824-1.232) | 0.939 | 0.729 (0.838-1.264) | 0.785 |
| Q4 | 1.022 (0.838-1.247) | 0.826 | 1.078 (0.881-1.319) | 0.468 | 1.104 (0.914-1.432) | 0.239 |
| Flavanones | - | - | - | - | - | - |
| Q1 | - | - | - | - | - | - |
| Q2 | 0.727 (0.672-0.892) | 0.431 | 0.822 (0.738-1.742) | 0.854 | 0.780 (0.672-1.483) | 0.746 |
| Q3 | 0.783 (0.679-1.032) | 0.557 | 0.839 (0.610-1.373) | 0.745 | 0.792 (0.653-1.642) | 0.165 |
| Q4 | 0.782 (0.670-1.298) | 0.642 | 0.934 (0.894-1.983) | 0.648 | 0.892 (0.783-1.832) | 0.617 |
| Flavones |  |  |  |  |  |  |
| Q1 | - | - | - | - | - |  |
| Q2 | 0.677 (0.562-1.321) | 0.522 | 0.894 (0.673-1.482) | 0.731 | 1.420 (0.893-1.835) | 0.875 |
| Q3 | 0.831 (0.781-1.370) | 0.483 | 0.790 (0.643-1.464) | 0.654 | 1.326 (0.864-1.721) | 0.763 |
| Q4 | 0.839 (0.801-1.394) | 0.341 | 1.013 (0.938-1.847) | 0.789 | 1.041 (0.831-1.673) | 0.648 |
| Anthocyanins |  |  |  |  |  |  |
| Q1 | - | - | - | - | - | - |
| Q2 | 0.851 (0.700-1.034) | 0.105 | 0.856 (0.703-1.042) | 0.122 | 0.862 (0.707-1.051) | 0.142 |
| Q3 | 0.793(0.648-0.969) | 0.023 | 0.825 (0.673-1.012) | 0.064 | 0.818 (0.665-1.006) | 0.057 |
| Q4 | 0.784 (0.644-0.956) | 0.016 | 0.808 (0.661-0.988) | 0.038 | 0.787 (0.637-0.971) | 0.026 |
| All-cause mortality | HR (95%CI) | p-value | HR (95%CI) | p-value | HR (95%CI) | p-value |
| Flavan-3-ols |  |  |  |  |  |  |
| Q1 | - | - | - | - | - | - |
| Q2 | 0.961 (0.740- 1.248) | 0.764 | 0.996 (0.765-1.296) | 0.973 | 0.959 (0.730-1.260) | 0.765 |
| Q3 | 0.936 (0.711-1.231) | 0.634 | 1.043 (0.790-1.377) | 0.765 | 0.991 (0.740-1.326) | 0.950 |
| Q4 | 0.789 (0.598-1.040) | 0.092 | 0.834 (0.631-1.101) | 0.200 | 0.739 (0.525-1.039) | 0.081 |
| Flavonols |  |  |  |  |  |  |
| Q1 | - | - | - | - | - | - |
| Q2 | 1.031 (0.839-1.342) | 0.634 | 0.981 (0.831-1.034) | 0.653 | 0.830 (0.781-1.343) | 0.521 |
| Q3 | 0.938 (0.738-1.333) | 0.499 | 0.830 (0.643-1.213) | 0.332 | 1.043 (0.830-1.442) | 0.403 |
| Q4 | 0.892 (0.830-1.384) | 0.320 | 0.988 (0.830-1.083) | 0.542 | 1.13 (0.991-1.423) | 0.129 |
| Flavanones |  |  |  |  |  |  |
| Q1 | - | - | - | - | - | - |
| Q2 | 1.381 (0.839-1.731) | 0.731 | 0.829 (0.782-1.672) | 0.483 | 0.894 (0.673-1.481) | 0.643 |
| Q3 | 0.993 (0.882-1.830) | 0.672 | 0.786 (0.892-1.731) | 0.321 | 0.763 (0.683-1.483) | 0.851 |
| Q4 | 0.982 (0.831-1.638) | 0.780 | 0.923 (0.838-1.857) | 0.430 | 0.883 (0.738-1.472) | 0.786 |
| Flavones |  |  |  |  |  |  |
| Q1 | - | - | - | - | - | - |
| Q2 | 0.882 (0.642-1.321) | 0.134 | 0.893 (0.738-1.310) | 0.524 | 0.734 (0.562-1.503) | 0.324 |
| Q3 | 0.673 (0.563-1.034) | 0.350 | 0.891 (0.638-1.072) | 0.452 | 0.634 (0.780-1.342) | 0.104 |
| Q4 | 0.831 (0.730-1.436) | 0.533 | 1.023 (0.809-1.382) | 0.133 | 0.891 (0.775-1.034) | 0.430 |
| Anthocyanins |  |  |  |  |  |  |
| Q1 | - | - | - | - | - | - |
| Q2 | 0.799 (0.609-1.047) | 0.104 | 0.840 (0.639-1.105) | 0.212 | 0.831 (0.632-1.094) | 0.187 |
| Q3 | 0.848 (0.649-1.108) | 0.226 | 0.913 (0.696-1.198) | 0.512 | 0.883 (0.671-1.161) | 0.372 |
| Q4 | 0.752 (0.573-0.988) | 0.040 | 0.826 (0.625-1.091) | 0.178 | 0.790 (0.591-1.057) | 0.112 |

Model 1: Age, Gender, BMI

Model 2: Age, Gender, BMI, Townsend Deprivation Index, Smoking-status, Education, Hypertension, Diabetes

Model 3: Age, Gender, BMI, Townsend Deprivation Index, Smoking-status, Education, Hypertension, Diabetes, Sodium intake, Coffee intake, Whole grains, Processed meat, Sugary drinks intake
